# Trends in Meeting Physical Activity Guidelines Among Urban and Rural Dwelling Adults — United States, 2008–2017

**DOI:** 10.15585/mmwr.mm6823a1

**Published:** 2019-06-14

**Authors:** Geoffrey P. Whitfield, Susan A. Carlson, Emily N. Ussery, Janet E. Fulton, Deborah A. Galuska, Ruth Petersen

**Affiliations:** 1 Division of Nutrition, Physical Activity and Obesity, National Center for Chronic Disease Prevention and Health Promotion, CDC.

Since the release of the 2008 Physical Activity Guidelines for Americans (https://health.gov/paguidelines/2008/pdf/paguide.pdf), the age-adjusted percentage of adults meeting the combined aerobic and muscle-strengthening guidelines increased from 18.2% to 24.3% in 2017 (*1*). Trends in urban and rural areas, across demographic subgroups, and among subgroups within urban and rural areas have not been reported. CDC analyzed 2008–2017 National Health Interview Survey (NHIS) data to examine trends in the age-standardized prevalence of meeting physical activity guidelines among adults aged ≥18 years living in urban and rural areas. Among urban and rural residents, prevalence increased from 19.4% to 25.3% and from 13.3% to 19.6%, respectively. Nationally, all demographic subgroups and regions experienced increases over this period; increases for several groups were not consistent year-to-year. Among urban residents, the prevalence was higher during 2016–2017 than during 2008–2009 for all demographic subgroups and regions. During the same period, prevalence was higher across all rural-dwelling subgroups except Hispanics, adults with a college education, and those living in the South U.S. Census region. Urban and rural communities can implement evidence-based approaches, including improved community design, improved access to indoor and outdoor recreation facilities, social support programs, and community-wide campaigns to make physical activity the safe and easy choice for persons of all ages and abilities (*2*–*4*). Incorporating culturally appropriate strategies into local programs might help address differences across subgroups.

Physical activity can lower a person’s risk for several chronic diseases, including coronary heart disease, stroke, obesity, and type 2 diabetes (*3*). To attain substantial health benefits, federal physical activity guidelines recommend that adults perform at least 150–300 minutes of moderate-intensity, or 75–150 minutes of vigorous-intensity aerobic physical activity per week, or an equivalent combination of moderate- and vigorous-intensity aerobic physical activity (i.e., the aerobic guideline) (*3*). In addition, adults should do muscle-strengthening activities of at least moderate intensity that involve all major muscle groups on ≥2 days per week (i.e., the muscle-strengthening guideline) (*3*).

NHIS is an annual, multistage probability sample of U.S. households designed to be representative of the civilian, noninstitutionalized U.S. population.* Among sampled adults, sample sizes ranged from 21,781 (2008) to 36,697 (2014); response rates ranged from 53.0% (2017) to 66.3% (2011). Adults reported the frequency and duration of vigorous- and light- or moderate-intensity leisure-time physical activities.^†^ The number of weekly minutes was calculated as the product of frequency (occurrences per week) and duration (minutes per occurrence). To match guidelines, the number of weekly minutes of vigorous-intensity physical activity was doubled and added to the number of weekly minutes of light- or moderate-intensity activity (*3*). Participants were classified as meeting the aerobic guideline if this total was at least 150 minutes per week. Adults also reported muscle-strengthening activities^§^ and were classified as meeting the muscle-strengthening guideline if they reported such activity on ≥2 days per week. Participants were classified as meeting the combined aerobic and muscle-strengthening guidelines if they met both the aerobic and muscle-strengthening guidelines as defined.

The annual, age-standardized prevalence of meeting the combined guidelines was calculated for each year.^¶^ Results were stratified by demographic characteristics (self-reported sex, age, race/ethnicity, and level of educational attainment), Census region of residence, and urban or rural residence (classified according to the U.S. Census Bureau definition) (*5*). Results for the racial/ethnic group “non-Hispanic other” are presented for reference purposes but were not interpreted because multiple races were combined and the sample sizes were small. Trends were assessed using age-adjusted logistic regression and orthogonal polynomial contrasts. When trends deviated from linearity, the best-fitting model was identified using sequential permutation tests in JoinPoint (version 4.7.0.0; National Cancer Institute)**; slopes from the selected model provided annual percentage point changes. To quantify doubly stratified changes over the period, the first 2 and last 2 years of data (i.e., 2008–2009 and 2016–2017) were combined, and prevalence of meeting the combined guidelines was estimated separately for urban and rural residents, stratified by demographic characteristics and region. Differences between periods were tested using adjusted Wald tests. Results with p-values <0.05 were considered statistically significant. Weighted analyses were performed in Stata (version 15; StataCorp) following NHIS analytic guidelines.

From 2008 to 2017, the age-standardized prevalence of meeting the combined physical activity guidelines increased 30.4% among urban residents (from 19.4% to 25.3%) and 47.4% among rural residents (from 13.3% to 19.6%) (Figure). The prevalence increased across all demographic subgroups, among residents of urban and rural areas, and in all Census regions (Table 1). The overall average annual percentage point change ranged from 0.3 (adults aged 45–64 years and those with some college education) to 0.7 (adults aged 25–34 years and those residing in the Northeast). Increases stalled in middle years overall and for several subgroups (women, adults aged 25–34 years, non-Hispanic whites, adults with at least some college education, urban residents, and adults in the Midwest and West). For example, among urban residents, the prevalence increased 1.1 percentage points per year from 2008 to 2010 (95% confidence interval [CI] = 0.3–2.0), followed by a nonsignificant 0.1 percentage point increase per year from 2010 to 2015 (95% CI = −0.2–0.4), then increased 1.6 percentage points per year from 2015 to 2017 (95% CI = 0.8–2.4).

**FIGURE:**
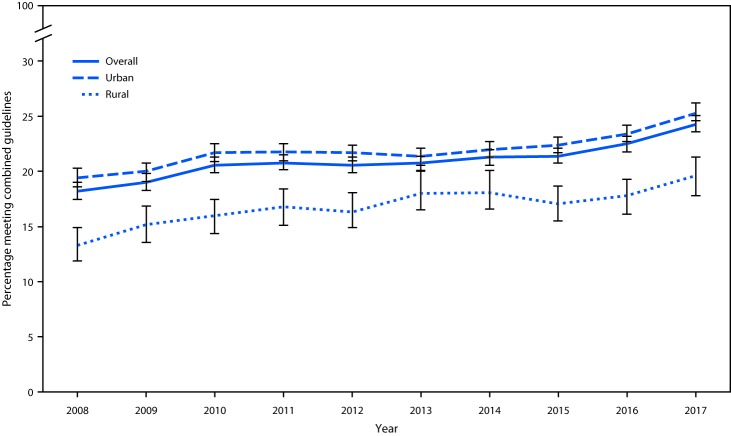
Age-standardized prevalence (with 95% confidence interval) of meeting the combined aerobic and muscle-strengthening physical activity guidelines among adults, by urban and rural residence — National Health Interview Survey, United States, 2008–2017

**TABLE 1:** Prevalence* of meeting the combined aerobic and muscle-strengthening physical activity guidelines, and prevalence trends among adults — National Health Interview Survey, United States, 2008, 2012, and 2017

Characteristic	% (95% CI)			Average APC 2008–2017 (95% CI)	Segment 1		Segment 2		Segment 3	
	2008	2012	2017		Years^†^	Segment average APC (95% CI)	Years^†^	Segment average APC (95% CI)	Years^†^	Segment average APC (95% CI)
**Total**	**18.2 (17.5 to 19)**	**20.6 (19.9 to 21.3)**	**24.3 (23.6 to 25.1)**	**0.5 (0.3 to 0.7)**	**2008–10**	**1.2 (0.4 to 2.0)**	**2010–15**	**0.2 (0.0 to 0.4)**	**2015–17**	**1.4 (0.6 to 2.2)**
**Sex**										
Men	21.7 (20.6 to 22.8)	24.3 (23.3 to 25.3)	28.8 (27.6 to 30.0)	0.6 (0.4 to 0.8)	2008–17	0.6 (0.4 to 0.8)	—	—	—	—
Women	14.9 (14.1 to 15.8)	17.1 (16.3 to 17.8)	20.1 (19.2 to 21.0)	0.4 (0.3 to 0.5)	2008–10	0.8 (0.2 to 1.4)	2010–15	0.2 (0.0 to 0.4)	2015–17	1.1 (0.5 to 1.7)
**Age group (yrs)**										
18–24	26.1 (23.6 to 28.8)	29.7 (27.5 to 31.9)	33.8 (31.1 to 36.7)	0.6 (0.4 to 1.0)	2008–17	0.6 (0.4 to 1.0)	—	—	—	—
25–34	22.6 (20.9 to 24.4)	26.6 (25.1 to 28.1)	30.6 (28.8 to 32.4)	0.7 (0.5 to 0.9)	2008–10	1.8 (0.3 to 3.3)	2010–15	0.3 (−0.1 to 0.7)	2015–17	1.7 (0.2 to 3.3)
35–44	19.4 (17.8 to 21.1)	21.7 (20.3 to 23.1)	27.5 (25.8 to 29.3)	0.5 (0.2 to 0.8)	2008–17	0.5 (0.2 to 0.8)	—	—	—	—
45–64	16.3 (15.2 to 17.4)	17.2 (16.2 to 18.2)	20.7 (19.6 to 21.8)	0.3 (0.2 to 0.5)	2008–15	0.2 (0.1 to 0.3)	2015–17	1.2 (0.1 to 2.3)	—	—
≥65	9.5 (8.3 to 10.8)	11.9 (10.9 to 12.9)	12.9 (11.9 to 13.9)	0.4 (0.3 to 0.4)	2008–17	0.4 (0.3 to 0.4)	—	—	—	—
**Race/Ethnicity**										
White, non-Hispanic	20.7 (19.7 to 21.7)	22.8 (21.9 to 23.7)	26.8 (25.9 to 27.8)	0.5 (0.3 to 0.7)	2008–10	1.0 (−0.1 to 2.2)	2010–15	0.1 (−0.2 to 0.5)	2015–17	1.7 (0.6 to 2.9)
Black, non-Hispanic	14.8 (13.3 to 16.4)	16.6 (15.2 to 18.0)	20.8 (18.8 to 23.0)	0.6 (0.4 to 0.8)	2008–17	0.6 (0.4 to 0.8)	—	—	—	—
Hispanic	11.3 (9.9 to 12.7)	15.4 (14.3 to 16.7)	18.7 (17.0 to 20.5)	0.6 (0.4 to 0.8)	2008–17	0.6 (0.4 to 0.8)	—	—	—	—
Other, non-Hispanic	15.3 (13.3 to 17.6)	19.0 (17.2 to 21)	22.6 (20.5 to 24.9)	0.6 (0.3 to 0.9)	2008–17	0.6 (0.3 to 0.9)	—	—	—	—
**Education**										
Less than high school	7.3 (6.2 to 8.7)	9.5 (8.3 to 10.8)	11.3 (9.7 to 13.2)	0.4 (0.3 to 0.5)	2008–17	0.4 (0.3 to 0.5)	—	—	—	—
High school	12.2 (11.1 to 13.4)	13.3 (12.3 to 14.5)	16.6 (15.3 to 18.0)	0.4 (0.2 to 0.6)	2008–17	0.4 (0.2 to 0.6)	—	—	—	—
Some college	19.9 (18.7 to 21.1)	21.7 (20.7 to 22.8)	23.9 (22.7 to 25.2)	0.3 (0.1 to 0.4)	2008–11	0.6 (0.1 to 1.1)	2011–15	−0.1 (−0.6 to 0.4)	2015–17	1.4 (0.3 to 2.4)
College graduate	27.9 (26.2 to 29.7)	31.2 (29.8 to 32.6)	33.9 (32.5 to 35.3)	0.4 (0.1 to 0.6)	2008–10	2.2 (0.9 to 3.6)	2010–13	−0.6 (−1.6 to 0.5)	2013–17	0.8 (0.5 to 1.1)
**Urban/Rural status**										
Urban	19.4 (18.6 to 20.3)	21.7 (21.0 to 22.4)	25.3 (24.5 to 26.2)	0.5 (0.3 to 0.7)	2008–10	1.1 (0.3 to 2.0)	2010–15	0.1 (−0.2 to 0.4)	2015–17	1.6 (0.8 to 2.4)
Rural	13.3 (11.9 to 14.9)	16.3 (14.6 to 18.1)	19.6 (18.0 to 21.3)	0.5 (0.3 to 0.7)	2008–17	0.5 (0.3 to 0.7)	—	—	—	—
**Census region**										
Northeast	18.2 (16.3 to 20.2)	20.3 (18.8 to 22.0)	25.6 (23.7 to 27.7)	0.7 (0.7 to 0.9)	2008–17	0.7 (0.7 to 0.9)	—	—	—	—
Midwest	19.9 (18.5 to 21.4)	21.5 (20.2 to 23.0)	25.9 (24.4 to 27.5)	0.4 (0.1 to 0.7)	2008–11	0.7 (0.3 to 1.1)	2011–15	−0.3 (−0.7 to 0.2)	2015–17	2.6 (1.7 to 3.5)
South	16.6 (15.3 to 17.9)	18.5 (17.4 to 19.6)	21.5 (20.3 to 22.8)	0.4 (0.3 to 0.6)	2008–17	0.4 (0.3 to 0.6)	—	—	—	—
West	19.0 (17.5 to 20.7)	23.2 (21.7 to 24.7)	26.4 (24.8 to 28.0)	0.5 (0.2 to 0.9)	2008–11	2.0 (0.8 to 3.2)	2011–15	−0.4 (−1.4 to 0.6)	2015–17	1.7 (−0.7 to 4.1)

**Abbreviations:** APC = annual percentage point change; CI = confidence interval.

* Age-standardized to the 2000 U.S. adult population, except age-specific estimates.

^†^ Segments were identified using JoinPoint software. Rows with only one segment indicate no statistically significant higher-order trends were present in JoinPoint (linear trend only). Subgroups with higher-order trends have information for either two or three segments, depending on which was the best fit in JoinPoint.

Among residents of urban areas, the prevalence of meeting the combined physical activity guidelines was higher overall during 2016–2017 (24.4%) than during 2008–2009 (19.8%), as well as across all demographic subgroups and in all Census regions (Table 2). Among rural residents, the prevalence increased across all demographic and regional subgroups except Hispanics (2008–2009 prevalence = 11.0%; 2016–2017 prevalence = 12.4%), adults with a college education (25.5%; 28.0%), and adults residing in the South Census region (13.2%; 14.7%).

**TABLE 2:** Prevalence* of meeting the combined aerobic and muscle-strengthening physical activity guidelines among urban and rural adult residents by selected demographic characteristics — National Health Interview Survey, United States, 2008–2009 and 2016–2017

Characteristic	Urban				Rural			
	2008–2009	2016–2017	Differences^†^		2008–2009	2016–2017	Differences^†^	
	% (95% CI)	% (95% CI)	Abs (95% CI)	Rel % (95% CI)	% (95% CI)	% (95% CI)	Abs (95% CI)	Rel % (95% CI)
**Total**	**19.8 (19.2–20.4)**	**24.4 (23.9–25.0)**	**4.7 (3.8–5.5)**	**23.6 (18.6–28.5)**	**14.3 (13.1–15.5)**	**18.7 (17.6–19.9)**	**4.4 (2.7–6.1)**	**31.0 (17.5–44.6)**
**Sex**								
Men	23.3 (22.5 to 24.2)	29.0 (28.1 to 29.9)	5.6 (4.4 to 6.9)	24.1 (18.0 to 30.1)	16.4 (14.9 to 18.1)	21.1 (19.4 to 22.8)	4.6 (2.3 to 7.0)	28.1 (11.9 to 44.4)
Women	16.4 (15.7 to 17.2)	20.2 (19.5 to 20.9)	3.7 (2.7 to 4.8)	22.8 (15.9 to 29.7)	12.1 (10.9 to 13.5)	16.3 (15.0 to 17.8)	4.2 (2.2 to 6.1)	34.3 (15.5 to 53.2)
**Age group (yrs)**								
18–24	27.1 (25.2 to 29.0)	33.4 (31.5 to 35.5)	6.4 (3.6 to 9.1)	23.5 (12.3 to 34.8)	18.0 (14.5 to 22.2)	25.3 (21.3 to 29.7)	7.2 (1.5 to 13.0)	40.2 (2.1 to 78.3)
25–34	24.1 (22.8 to 25.4)	31.3 (29.8 to 32.7)	7.2 (5.2 to 9.1)	29.8 (20.5 to 39.1)	19.2 (16.3 to 22.5)	23.6 (20.9 to 26.5)	4.4 (0.2 to 8.6)	22.8 (−1.7 to 47.4)
35–44	21.6 (20.4 to 22.9)	26.6 (25.3 to 27.9)	5.0 (3.1 to 6.8)	23.0 (13.5 to 32.5)	15.8 (13.5 to 18.4)	21.5 (18.9 to 24.4)	5.7 (2.1 to 9.4)	36.3 (8.9 to 63.8)
45–64	17.8 (16.9 to 18.8)	20.9 (20.0 to 21.8)	3.1 (1.8 to 4.4)	17.2 (9.2 to 25.1)	12.8 (11.3 to 14.3)	16.1 (14.8 to 17.6)	3.4 (1.3 to 5.4)	26.5 (8.0 to 44.9)
≥65	10.7 (9.7 to 11.9)	13.8 (13.0 to 14.7)	3.1 (1.7 to 4.4)	28.7 (13.8 to 43.5)	7.0 (5.8 to 8.4)	9.5 (8.4 to 10.7)	2.6 (0.8 to 4.3)	36.6 (6.2 to 67.1)
**Race/Ethnicity**								
White, non-Hispanic	23.1 (22.2 to 23.9)	27.8 (27.1 to 28.6)	4.8 (3.6 to 5.9)	20.7 (15.1 to 26.2)	14.7 (13.5 to 16.1)	19.5 (18.2 to 20.8)	4.7 (2.9 to 6.5)	32.1 (17.8 to 46.4)
Black, non-Hispanic	17.0 (15.8 to 18.3)	21.1 (19.6 to 22.7)	4.1 (2.1 to 6.1)	24.0 (11.0 to 37.0)	10.3 (7.7 to 13.6)	17.9 (13.1 to 24.1)	7.7 (1.5 to 13.9)	74.8 (1.9 to 147.6)
Hispanic	12.1 (11.1 to 13.1)	18.1 (16.9 to 19.4)	6.0 (4.4 to 7.6)	49.8 (33.6 to 66.0)	11.0 (7.7 to 15.6)	12.4 (8.8 to 17.3)	1.4 (−4.4 to 7.1)	12.5 (−42.8 to 67.8)
Other, non-Hispanic	15.0 (13.5 to 16.6)	21.0 (19.5 to 22.7)	6.0 (3.8–8.2)	40.1 (22.3 to 57.9)	13.3 (9.1 to 19.1)	15.8 (12.4 to 20.0)	2.5 (−3.8 to 8.7)	18.4 (−33.9 to 70.7)
**Education**								
Less than high school	7.7 (6.8 to 8.7)	11.4 (10.1 to 12.8)	3.7 (2.1 to 5.3)	47.7 (23.3 to 72.2)	5.8 (4.1 to 8.2)	10.6 (8.1 to 13.8)	4.8 (1.3 to 8.2)	82.0 (2.8 to 161.1)
High school	12.5 (11.7 to 13.4)	16.3 (15.2 to 17.4)	3.8 (2.4 to 5.2)	30.1 (17.4 to 42.8)	9.9 (8.5 to 11.6)	12.2 (10.7 to 13.9)	2.3 (0.1 to 4.5)	22.8 (−2.1 to 47.7)
Some college	21.6 (20.6 to 22.6)	24.1 (23.1 to 25.0)	2.5 (1.1 to 3.9)	11.7 (4.9 to 18.4)	16.0 (14.2 to 18.0)	20.4 (18.6 to 22.2)	4.4 (1.7 to 7.0)	27.2 (8.3 to 46.0)
College graduate	29.6 (28.2 to 31.0)	33.9 (32.8 to 35.0)	4.3 (2.6 to 6.1)	14.6 (8.1 to 21.1)	25.5 (22.5 to 28.7)	28.0 (25.4 to 30.8)	2.5 (−1.6 to 6.6)	9.9 (−7.2 to 27.0)
**Census region**								
Northeast	18.7 (17.2 to 20.4)	24.9 (23.5 to 26.2)	6.1 (4.0 to 8.2)	32.7 (19.3 to 46.1)	16.4 (13.3 to 20.1)	24.2 (21.2 to 27.6)	7.8 (3.1 to 12.4)	47.4 (11.4 to 83.4)
Midwest	21.8 (20.5 to 23.1)	25.7 (24.4 to 27.1)	3.9 (2.1 to 5.8)	18.1 (8.9 to 27.3)	14.1 (12.7 to 15.7)	19.9 (18.0 to 22.1)	5.8 (3.3 to 8.4)	41.2 (20.3 to 62.1)
South	18.9 (17.8 to 20.0)	22.5 (21.5 to 23.5)	3.6 (2.1 to 5.1)	19.2 (10.6 to 27.8)	13.2 (11.5 to 15.0)	14.7 (13.2 to 16.3)	1.5 (−0.9 to 3.9)	11.3 (−7.8 to 30.5)
West	19.9 (18.6 to 21.2)	25.7 (24.7 to 26.9)	5.9 (4.2 to 7.6)	29.6 (19.7 to 39.6)	15.9 (12.1 to 20.6)	25.4 (21.2 to 30.2)	9.6 (3.4 to 15.7)	60.1 (9.0 to 111.3)

**Abbreviations:** Abs = absolute difference; CI = confidence interval; Rel = relative difference.

* Age-standardized to the 2000 U.S. adult population, except for age-specific estimates.

^†^ Absolute difference confidence intervals that exclude 0 indicate statistically significant differences.

## Discussion

Since release of the 2008 Physical Activity Guidelines for Americans, the prevalence of meeting the combined aerobic and muscle-strengthening physical activity guidelines among adults has increased in both urban and rural areas. Despite the increases, additional progress is needed. In 2017, only one in four (25.3%) urban residents and one in five (19.6%) rural residents met the combined guidelines. To continue and perhaps accelerate progress, communities can implement evidence-based approaches that make physical activity the safe and easy choice, including improvements to community design, improved access to indoor and outdoor recreation facilities, social support programs, and community-wide campaigns (*2*–*4*).

The prevalence of meeting the combined guidelines tended to be lower among rural residents than among urban residents, and remains below the national target established in Healthy People 2020 (20.1%). Environmental differences might contribute to this finding. For example, environmental supports and nearby destinations including sidewalks, public transit, and shops can encourage physical activity, but are less common in rural than in urban areas (*6*). To help rural communities address these challenges, the Federal Highway Administration published Small Town and Rural Multimodal Networks, a 2016 design guide with illustrated examples of activity-friendly infrastructure.^††^ Additionally, rural communities might have existing, underused supports for aerobic and muscle-strengthening activities, such as schoolyards, parks, or community centers. Improving access to and awareness of existing facilities through shared-use agreements, facility improvements, and outreach or community-wide campaigns could be effective strategies for rural communities (*3*,*4*).

The lack of improvement from 2008–2009 to 2016–2017 among rural Hispanics and adults living in the South is notable and concerning because of demonstrated burdens of obesity, diabetes, and related comorbidities in these groups (*7*). CDC’s Racial and Ethnic Approaches to Community Health program helps communities implement culturally appropriate programs to address health issues among minority populations.^§§^ Under this program, the health authority in Cabarrus County, North Carolina initiated work with local organizations to improve community design and implement shared-use agreements with schools and churches in predominantly Hispanic and African-American areas. Similarly, CDC’s High Obesity Program works with state universities to improve physical activity in counties with high obesity prevalence, often in the rural South.^¶¶^ For example, Martin County, Kentucky recently increased opportunities for physical activity with a walking trail linking housing to nearby destinations in the small town of Warfield. These programs might serve as examples for other communities to follow.

The increases documented in this report are encouraging as they demonstrate that population-level change is possible, but additional progress is needed. To continue and perhaps accelerate progress, CDC launched Active People, Healthy Nation, which aims to improve the physical activity levels of 27 million Americans over 10 years (*8*). This multisector initiative presents five action steps, including 1) delivering programs that work, 2) mobilizing partners to ensure that physical activity initiatives are prioritized, coordinated, and updated using research and evaluation findings; 3) sharing messages that promote active lifestyles; 4) training leaders to take action and encourage both sector-specific and cross-sector training; and 5) developing technologies and tools to help address gaps in physical activity-related data. Active People, Healthy Nation provides a comprehensive path to improving physical activity levels in the United States and is poised to continue the momentum documented here.

The findings in this report are subject to at least three limitations. First, the physical activity assessment in NHIS is limited to leisure-time physical activity. Residents of rural areas might accrue more physical activity through occupational or domestic tasks than do residents of urban areas (*9*), although this might be somewhat offset by less transportation-related activity among rural residents (*10*). Second, NHIS asks about participation in light-intensity and moderate-intensity activity in a single question, which likely overestimates prevalence estimates of meeting the aerobic guideline, which focuses on activities of at least moderate intensity. Finally, all data are based on self-reports and might overestimate physical activity because of social desirability biases.

Despite recent increases in meeting physical activity guidelines, insufficient participation in physical activity remains a public health concern. By focusing on evidence-based approaches and the action steps of Active People, Healthy Nation, communities in both urban and rural areas can make physical activity the safe and easy choice for all U.S. residents.

**Summary**

**What is already known about this topic?**

The prevalence of meeting the combined aerobic and muscle-strengthening physical activity guidelines among adults increased since 2008 but remained low (24.3%) in 2017.

**What is added by this report?**

Since 2008, the prevalence of meeting physical activity guidelines increased from 19.4% to 25.3% among urban residents and from 13.3% to 19.6% among rural residents. Among urban residents, all subgroups reported increases, whereas among rural residents, no increases were reported among Hispanics and adults living in the South.

**What are the implications for public health practice?**

Despite increases, physical activity prevalence remains low, especially for some rural subgroups with high incidences of chronic diseases. Incorporating culturally appropriate strategies into local, evidence-based programs might help communities build on recent progress.

## Footnotes

*
https://www.cdc.gov/nchs/nhis/index.htm .

† Leisure-time physical activity prompt: “The next questions are about physical activities (exercise, sports, physically active hobbies...) that you may do in your LEISURE time.” Frequency of vigorous-intensity activity: “How often do you do vigorous leisure-time physical activities for at least 10 minutes that cause heavy sweating or large increases in breathing or heart rate?” Duration: “About how long do you do these vigorous leisure-time physical activities each time?” Frequency of light to moderate intensity activity: “How often do you do light or moderate leisure-time physical activities for at least 10 minutes that cause only light sweating or a slight to moderate increase in breathing or heart rate?” Duration: “About how long do you do these light or moderate leisure-time physical activities each time?”

§ Frequency of muscle-strengthening activity: “How often do you do leisure-time physical activities specifically designed to strengthen your muscles such as lifting weights or doing calisthenics?”

¶ Estimates were age-adjusted using the 2000 U.S. population as the standard population and using five age groups: 18–24, 25–34, 35–44, 45–64, and ≥65 years.

**
https://surveillance.cancer.gov/joinpoint/ .

††
https://www.fhwa.dot.gov/environment/bicycle_pedestrian/publications/small_towns/ .

§§
https://www.cdc.gov/nccdphp/dnpao/state-local-programs/reach/index.htm .

¶¶
https://www.cdc.gov/nccdphp/dnpao/state-local-programs/hop-1809/high-obesity-program-1809.html .
